# Biomimetic surface structuring using cylindrical vector femtosecond laser beams

**DOI:** 10.1038/srep45114

**Published:** 2017-03-22

**Authors:** Evangelos Skoulas, Alexandra Manousaki, Costas Fotakis, Emmanuel Stratakis

**Affiliations:** 1 Institute of Electronic Structure and Laser (IESL), Foundation for Research and Technology (FORTH), N. Plastira 100, Vassilika Vouton, 70013, Heraklion, Crete, Greece; 2 Materials Science and Technology Department, University of Crete, 71003 Heraklion, Greece; 3 Physics Department, University of Crete, 71003 Heraklion, Greece

## Abstract

We report on a new, single-step and scalable method to fabricate highly ordered, multi-directional and complex surface structures that mimic the unique morphological features of certain species found in nature. Biomimetic surface structuring was realized by exploiting the unique and versatile angular profile and the electric field symmetry of cylindrical vector (CV) femtosecond (fs) laser beams. It is shown that, highly controllable, periodic structures exhibiting sizes at nano-, micro- and dual- micro/nano scales can be directly written on Ni upon line and large area scanning with radial and azimuthal polarization beams. Depending on the irradiation conditions, new complex multi-directional nanostructures, inspired by the *Shark’s* skin morphology, as well as superhydrophobic dual-scale structures mimicking the *Lotus’* leaf water repellent properties can be attained. It is concluded that the versatility and features variations of structures formed is by far superior to those obtained via laser processing with linearly polarized beams. More important, by exploiting the capabilities offered by fs CV fields, the present technique can be further extended to fabricate even more complex and unconventional structures. We believe that our approach provides a new concept in laser materials processing, which can be further exploited for expanding the breadth and novelty of applications.

Nature has always provided a plethora of functional surfaces exhibiting unique, complex hierarchical morphologies with dimensions of features ranging from the macroscale to the nanoscale. Such morphologies are always behind the superior properties exhibited by the natural surfaces, including extreme wetting, floatation, adhesion, friction and mechanical strength^1^. In this context, the design and the fabrication of biomimetic structures is of significant importance and provides a virtually endless potential for the development of novel artificial materials and systems.

Despite the increasing scientific interest, the complex structure of most of the natural surfaces has been proven to be extremely difficult to mimic. Several fabrication techniques have been developed, based on top-down and bottom-up processing schemes^1^. Among those, direct laser structuring is a material independent and versatile technique that presents some key benefits for precise surface modification, over competitive techniques^2^. In particular, laser processing with fs pulses offers advantages in minimizing thermal effects and collateral damage, allowing localized modifications with a large degree of control over the shape, size and the range of features that can be produced. Indeed, fs laser induced surface structuring has been demonstrated to produce numerous biomimetic structures^2^,^3^,^4^,^5^ for a range of applications, including microfluidics^3^,^5^,^6^, tribology^7^,^8^,^9^, tissue engineering^2^,^10^ and advanced optics^11^.

A prominent aspect of the fs laser material interaction is that the spatial features of the surface structures attained are strongly correlated with the laser beam polarization. This is for example the case of laser-induced periodic surface structures (LIPSS) and quasi-periodic microgrooves, which are preferentially oriented perpendicular and parallel to the laser polarization respectively^12^,^13^,^14^,^15^,^16^,^17^. However, to date, laser fabrication of biomimetic structures has been demonstrated using laser beams with a Gaussian intensity spatial profile and spatially homogeneous linear polarization^18^. In this context and based on the sensitivity of laser induced structures on laser polarization, it is possible to further advance the complexity of the fabricated structures via utilizing laser beams with a spatially inhomogeneous state of polarization^19^,^20^,^21^,^22^. CV beams, exhibiting tangential polarization states, are prominent examples^23^,^24^,^25^,^26^,^27^,^28^,^29^.

In this paper, we report on the direct fs laser biomimetic surface structuring via the use of CV beams generated by an s-waveplate^30^, which transforms a linearly polarized Gaussian beam to a CV beam with vectorial polarization states. It is shown that dynamic surface processing with radial and azimuthal polarization beams, gives rise to large areas of complex biomimetic structures. In particular, the formation of bioinspired multi-directional periodic structures that mimic the sharks’ skin morphology, as well as that of well-ordered superhydrophibic hierarchical micro/nano structures on Ni surfaces, is demonstrated. Although the fabrication of these particular morphologies was demonstrated, the versatility and features’ variations of attainable structures could be practically endless. We believe that our approach brings about a new thinking in laser processing of materials and can be further extended to provide even more complex biomimetic structures for numerous potential applications.

## Materials and Methods

**Figure 1:**
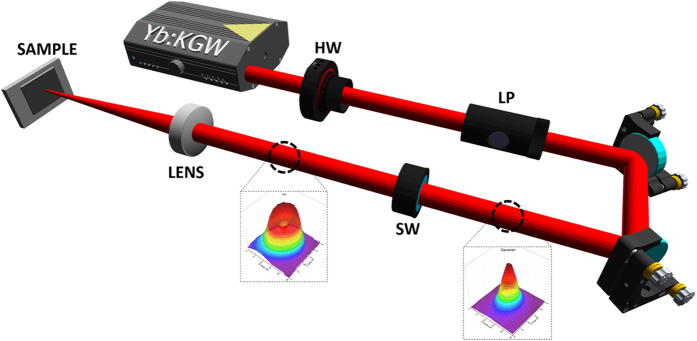
Schematic of the experimental setup developed for the laser induced fabrication of biomimetic structures. The incident laser fluence was varied by means of a λ/2 waveplate (HW). The Gaussian profile emitted by the laser source is transformed to a CV beam using a rotating s-waveplate (SW). Depending on the SW rotation angle, the CV beam polarization can be changed from radial to azimuthal respectively.

The experimental apparatus used to fabricate biomimetic surface structures using CV beams is presented in Fig. 1. Commercially available polished Ni films of 99.9% purity and average thickness of 100 μm where used as samples. The Yb:KGW laser source produced linearly polarized pulses of 170fs, 1 KHz repetition rate and 1026 nm central wavelength. CV beams of radial and azimuthal polarisation, exhibiting a donut-shaped profile, were generated by means of an s-waveplate. The characteristics of the produced CV beams are presented in Fig. 1S. Following the s-waveplate, the CV beam was focused on the sample via an achromatic convex lens of 60 mm focal length while the Gaussian spot diameter, measured by a CCD camera on the focal plane at 1/e^2^, was 32 μm. Samples were fixed onto a 3-axis motorized stage and positioned perpendicular to the incident beam. All irradiations were performed in ambient environment. Due to the different spatial profile of the Gaussian with respect to an CV beam, fluence, *φ* calculations were made separately in each case as shown at refs 28,29. At the same time, the incident number of pulses, *NP*, was controlled by an electromagnetic beam shutter.

For the dynamic processing experiments, line or area scans were produced at variable velocity values, *v*, ranging from 0.3 mm/s to 2.0 mm/s. In this case, the effective number of laser pulses (*N*_*eff*_) per unit length or area should be determined respectively. *N*_*eff*_ has been commonly used for Gaussian beams and corresponds to the number of laser pulses falling, upon one-dimensional scanning, onto a length interval equal to the Gaussian beam diameter 2*w*_0_. In the case of CV beams, *N*_*eff*_ should depend on the corresponding donut shaped beam area. For line scanning at constant velocity *v* and at repetition rate *f* and assuming donut diameters *R* (outer), *r* (inner), the effective pulse number *Neff*_*line*_ can be defined as:


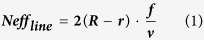


While for large area scanning at constant velocity *v*, at repetition rate *f* and individual line separation *δ, Neff*_*area*_ can be defined as:


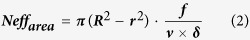


While, the spot overlap area is defined as:


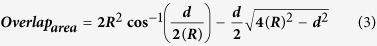



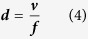


where *d* is the distance between two consecutive circular spot centers.

The morphology of the laser-induced structures has been characterized by scanning electron microscopy (JEOL JSM-7500F). While, the structures’ characteristics was determined by two dimensional fast Fourier transform (2D-FFT) analysis of the respective SEM images using the Gwyddion software. Details for the periodicity calculation can be found in the supporting information Fig. 2S. For higher accuracy and error estimation, SEM images of three spots, produced with identical conditions were statistically analyzed.

The wetting properties of the fabricated surfaces were measured by the sessile droplet method, performed using the DataPhysics OCA 20 system. In particular, distilled water drops of 4 μl were deposited on each surface tested and the average value of the water contact angle (CA), obtained from five measurements as well as the standard deviation, was calculated.

## Results and Discussion

### Single and multiple shot irradiation experiments

**Figure 2:**
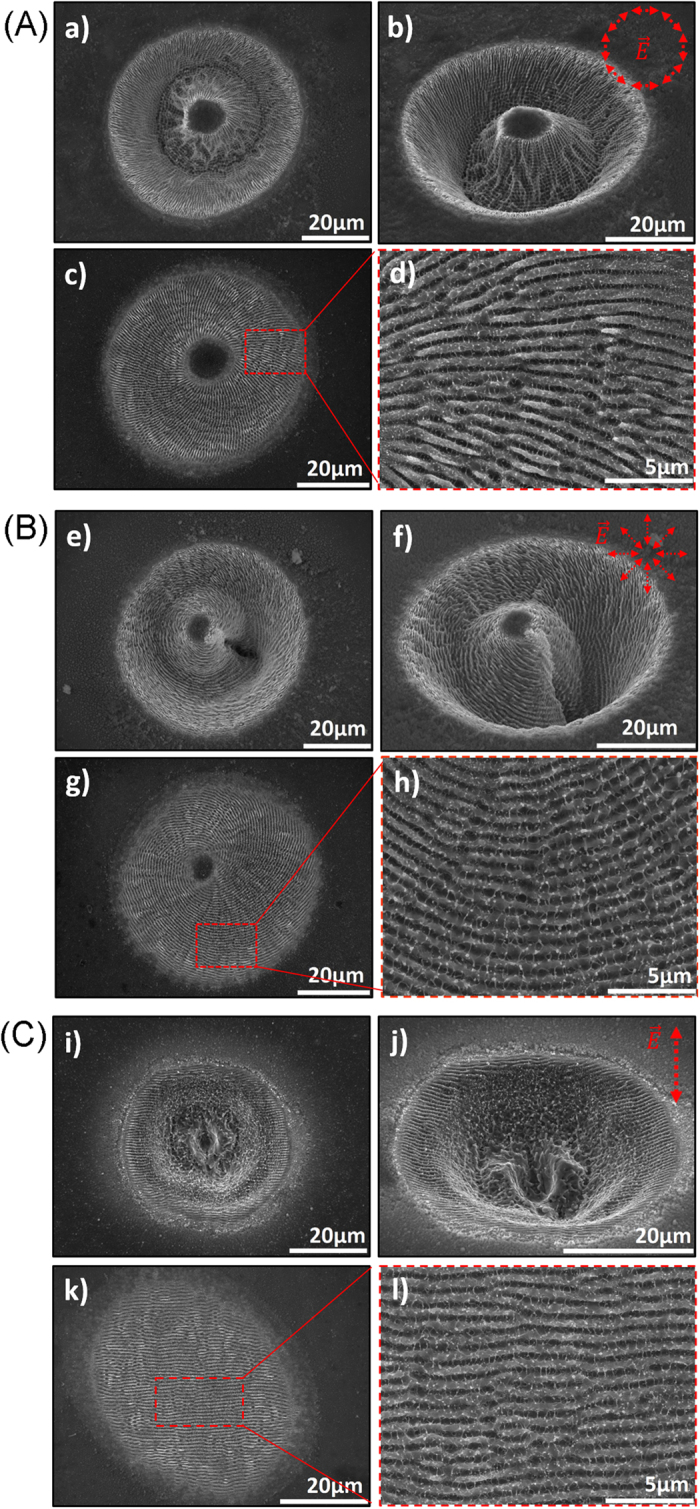
SEM images of fs laser-induced structures formed on Ni surfaces upon irradiation with azimuthal (a–d), radial (e–h) and linear (i–l) polarization beams respectively, using *φ* = 0.24 J/cm^2^ and *NP* = 1000 (a,b,e,f,i,j) or *NP* = 100 (c,d,g,h,k,l). All pictures show top-views, except (b), (f), (j) that present 45-degrees views. Images (d), (h) and (l) are higher magnifications of the red-dashed-square areas.

**Figure 3:**
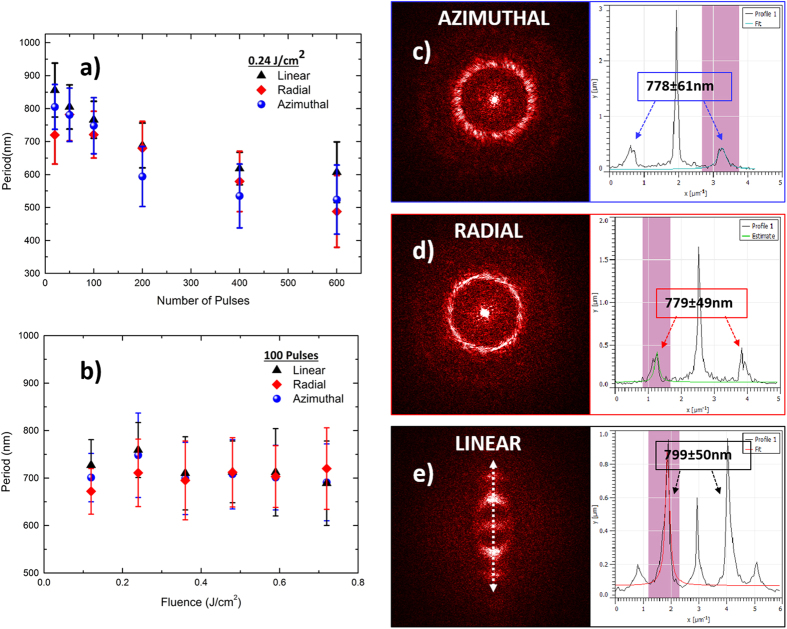
LIPSS dependence on (**a**) the fluence and (**b**) the number of pulses for the azimuthal, radial and linear beams respectively. On the right side the 2D-FFT analysis corresponding to the SEM images (**c**), (**g**) and (**k**) of Fig. 2 is demonstrated. Specifically, the 2D-FFT image spectra are presented for each polarization, together with the respective line profiles obtained via image cross-sections. The corresponding calculated LIPSS periodicity is shown in the inset.

In a first step, the characteristics of laser-induced structures formed upon variation of the *NP* (1–1000 at a constant fluence of 0.24 J/cm^2^), as well as the incident fluence (0.17 J/cm^2^–0.74 J/cm^2^, at a constant *NP* = 100) of the CV beams, was investigated. SEM imaging of the respective spots indicated that for *NP* < 5 no periodic structures were formed in the whole range of fluences used. While, from 2 ≤ *NP* ≤ 5, surface roughness was significantly increased and resembles a nanostructured grating with a tendency to orientate parallel to the incident polarization (Fig. 3S). For higher *NP*, the resulting surface comprises a central microstructure formed in the inner region of the CV beam, exhibiting almost null intensity, while the donut area was always decorated with a characteristic texture of LIPSS, always aligned perpendicular to the laser polarization. Accordingly, LIPSS produced with azimuthally polarized light showed radial spatial distribution, while LIPSS obtained with radial polarization exhibited a concentric ring spatial distribution. Figure 2 presents typical examples of SEM images of such structures for Gausian linear, CV radial and azimuthal polarization respectively at specific irradiation conditions. The results from the parametric analysis, described above, showed that, regardless the polarization condition, the LIPSS periodicity decreases with *NP*s, while it is weakly influenced by the incident fluence (Fig. 3). At the same time, the crater depth and thus the height of the microstructure formed at the spot center of the CV beam can be changed, upon increasing *NP* and/or laser fluence, in the range from hundreds of nanometers to a few tens of microns.

As shown in Fig. 3, the LIPSS period progressively decreases for 5 < *NP* < 600, with a trend to saturate at higher *NPs,* an effect which is valid for both Gaussian (linear polarization in Fig. 3) and CV beams. On the contrary, LIPSS period is weakly dependent on the incident fluence, despite the spatial profile and polarization of the beam. This behavior has been recently addressed by our group^31^, via a combined theoretical and experimental study, showed that it can be attributed to a synergy of electrodynamic and hydrodynamical effects^13^.

Figure 3(c–e) presents also the 2D-FFT analysis used to determine the ripples periodicity, performed on typical SEM images of spots created by linear Gaussian, radial and azimuthal CV beams respectively. It can be observed that the 2D-FFT image characteristics reveal the polarization type, as it exhibits a preferential directionality for linear, a radial distribution for azimuthal and a vortex-like pattern for radial polarization respectively.

### Line scans using CV beams

**Figure 4:**
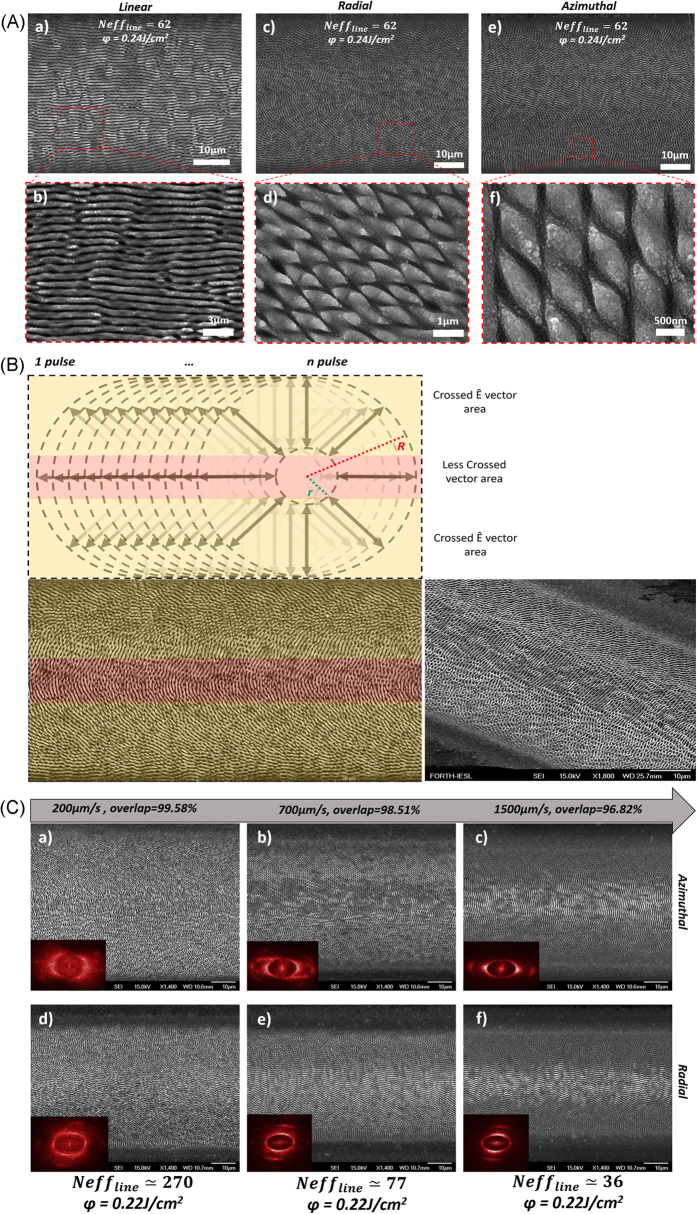
(**A**) Top-view SEM images depicting line scans produced by linearly (a,b), radially (c,d), and azimuthally polarized (e,f) beams, respectively, at *v* = 0.5 mm/s (*Neff*_*line*_ = 62), and *φ* = 0.24 J/cm^2^. The images (b,d,f) are higher magnifications of an area inside the red-dashed-squares and reveal the biomimetic shark skin-like morphology of the processed areas; (**B**) Schematic of the beam overlap process during a linescan with a CV beam; a typical linescan SEM image is shown for comparison; (**C**) SEM images of linescans produced by azimuthally (a,b,c) and radially (d,e,f) polarized CV beams of a constant fluence, *φ* = 0.22 J/cm^2^, at different scanning speeds. The corresponding 2D-FFT images are shown as insets.

Following spot analysis, line processing experiments were performed in scanning mode, using different scan velocities (thus *N*_*eff,line*_, calculated by Eq. 2, Materials and Methods section) and spot overlap at a constant *φ* value. Figure 4A exemplifies the characteristic surface morphologies attained, in top-view SEM micrographs of scanned lines, obtained at *v* = 0.5 mm/s (*Neff*_*line*_ = 62) and *φ* = 0.24 J/cm^2^, for linear Gaussian (4A-a, b), radial (4A-c, d) and azimuthal (4A-e, f) CV beams respectively. Contrary to the, mostly applied to date, linear polarization case, dynamic surface processing with CV beams produces multi-directional rhombus-like structures exhibiting a radial or azimuthal directionality respectively. Such rhombus-like structures significantly mimic the placoid structures found in the skin of shark, although their characteristic size differs substantially from those comprising the shark skin^8^,^32^,^33^.

To shed light on the creation of such novel structures, the line scan areas were carefully examined by SEM imaging. As presented in Fig. 4B, it was revealed that the central part of the scan lines is patterned with LIPSS exhibiting a radial or azimuthal orientation (see Fig. 2), depending on the CV beam used for scanning. While, the linescan peripheral areas are textured with the shark skin-like rhomboid structures. As also shown in Fig. 4B, such structures become as the natural outcome of the overlap between successive CV beam spots, specifically as the beam advances on the surface during scan, the pulse overlapping effect leads to crossed vector peripheral areas, giving rise to the rhombic-shaped structures.

According to the above, the scanning speed that determines the degree of overlap (calculated by Eq. 3, Materials and Methods section should play significant role on the patterns’ morphology attained. This is evident in Fig. 4C, depicting SEM images of line scans, fabricated at variable scan velocities. The insets represents the corresponding 2D-FFT images for each line scan, showing that, as the spot overlap is increased, additional spatial frequencies are generated. It is clear that the scan speed significantly affects the complexity and thus the variety of the structures attained.

### Fabrication of large areas using CV beams

**Figure 5:**
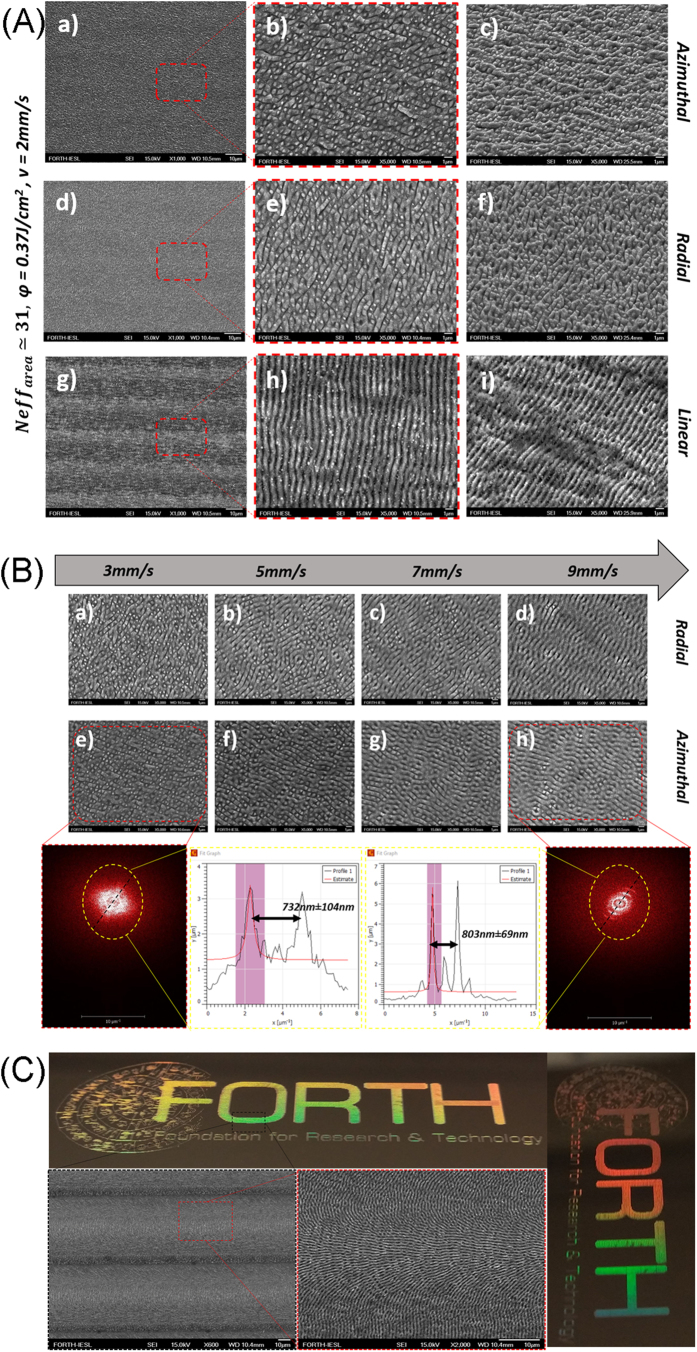
(**A**) SEM images from 16 mm^2^ areas fabricated using azimuthal (a–c), radial (d–f) and linear (g–i) polarization fabricated at *v* = 2 mm/s (*Neff*_*line*_ = 31), and *φ* = 0.37 J/cm^2^. All pictures show top-views, except (c), (f) and (i) that present 45-degrees views. Images (b), (d) and (h) are higher magnifications of the red-dashed-square areas. (**B**) SEM images of area scans produced by radially (a–d) and azimuthally (e–h) polarized CV beams of a constant fluence, *φ* = 0.37 J/cm^2^, at different scanning speeds. The corresponding 2D-FFT images for the lower and the higher scanning speed are shown, together with the respective 2D-FFT line profiles. The characteristic period of the ripple-like structures are also presented; (**C**) Structural coloration exhibited by a large area pattern, depicting the FORTH logo, fabricated with a azimuthally polarized CV beam of *φ* = 0.36 J/cm^2^ at *v* = 1 mm/s (*Neff*_*line*_ = 30).

The complexity of the structures attained can be further enriched upon areal scans with CV beams. Figure 5A (a–h) presents SEM images of 16 mm^2^ areas, fabricated at *φ* = 0.37 J/cm^2^, *v* = 2 mm/s and *Neff*_*area*_ = 31, using linear Gaussian, radial and azimuthal beams respectively. It can be observed that the structures attained are multi-directional with no preferential shape and orientation. Such surface morphology occurs due to the overlap between adjacent spots during the scanning process. Accordingly, the scanning speed together with the overlap between adjacent spots, are the most important parameters affecting the morphology of the surfaces attained. The effect of the scanning speed is presented in Fig. 5B showing large areas fabricated using azimuthally polarized CV beam, at different scanning velocities. Morphological and 2D-FFT imaging analyses reveal that when the scanning speed is high (low *Neff*_*area*_), the structures showed higher periodicity and a more linearly arranged orientation, closer to that observed upon processing with linearly polarized beams. On the contrary the structures fabricated with high *Neff*_*area*_ (i.e. at low speeds), are characterized by many spatial frequencies; such morphologies could not be attained upon processing with linearly polarized fs beams.

The physical properties of the unique surface morphologies attained are yet to be examined and further work, in this direction, is under progress. Wettability characterization of such surfaces showed only a small increase in hydrophobicity, compared with the non-irradiated, one. At the same time, some of the surfaces exhibited a remarkable coloration, attributed to light diffraction at the periodic structures of the laser treated area. An example of such coloration is presented in Fig. 5C, showing the, laser-marked, FORTH logo with a size of 5.1 cm × 1.8 cm. The exact optical properties of such unique structures are, however, under investigation.

The above findings suggest that surface processing with ultrashort CV beams could be a novel approach to increase the complexity of the structures attained and thus further advance the capabilities of ultrashort pulsed laser processing.

### Fabrication of large areas of hierarchical structures

**Figure 6:**
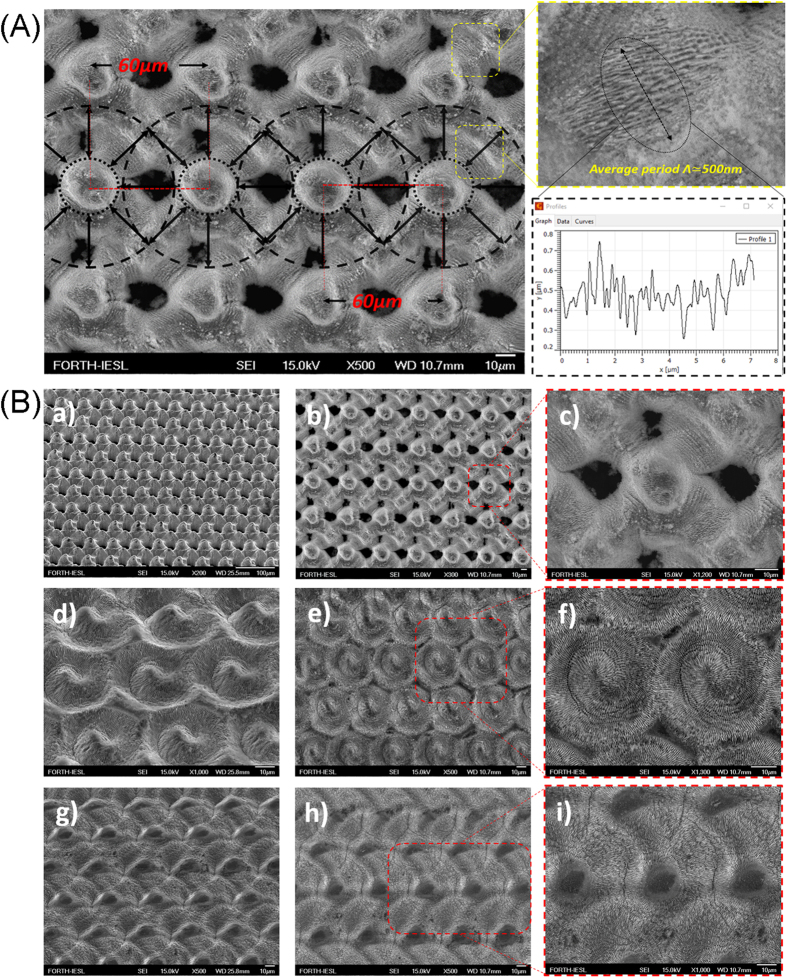
(**A**) Top-view SEM image of a surface fabricated via spot-by-spot processing 500 radially polarized pulses of 9.45 J/cm^2^ per spot; the dashed lines present the corresponding beam overlap upon spot-by-spot processing; the submicron sized rippled-like secondary structure decorating the primary microstructures is shown on the right, together with a respective line profile used to calculate the periodicity; (**B**) SEM images of three types of dual-scale surfaces fabricated via spot-by-spot processing with CV beams; (a,b) show low- and high- magnification 45° views of HR surfaces fabricated with 500 radially polarized pulses of 9.45 J/cm^2^ per spot; (c) shows the top view of a single microstructure of the HR surface; (d,e) show low- and high- magnification 45° views of MR surfaces fabricated with 400 radially polarized pulses of 1.12 J/cm^2^ per spot; (f) shows the top view of a single microstructure of the MR surface; (g,h) show low- and high- magnification 45° views of LR surfaces fabricated with 600 azimuthally polarized pulses of 0.42 J/cm^2^ per spot; (i) shows the top view of a single microstructure of the LR surface.

A multi-scale structuring approach is especially important in mimicking natural surfaces comprising structures with morphology at multiple length scales. In this respect, the fabrication of precisely controlled surfaces consisting of structures with more than one spatial frequency, is desirable. As mentioned above and presented in Fig. 2, the crater profiles corresponding to multiple pulse irradiation with CV beams, exhibit a primary microstructure at the center decorated with secondary submicron ripples. This morphology could provide a template motif for the fabrication of dual-scale, high- and low- spatial frequency structures. In a first step, a sequence of CV spots (Fig. 6) was used to fabricate a periodic array of microcones (the height of which, can be varied upon changing the incident φ), complemented by LIPSS (the periodicity of which can be tuned, in the range 1000–400 nm, upon changing the incident *NP*). At the same time, the distance among microstructures can be varied upon changing the overlap between adjacent spots. Using such a spot by spot process, areas of several millimeters square were fabricated. A typical example is presented in Fig. 6, showing a top-view SEM image of an array of microcones, decorated with periodic ripples of average periodicity of about 500 nm, produced by a series of radially polarized spots repeated over the whole area with overlap of 15.9% between two concecutive spots.

Using this approach, various complex surfaces can be produced upon different combinations of φ and NP, using radial and azimuthal CV beams respectively. Typical examples are presented in Fig. 6, however the variety of dual-scale biomimetic surfaces that can be produced is practically endless. Based on the sharpness of the principal microstructure, such surfaces can be classified to be of high (HR) (Fig. 6B,a,b), medium (MR) (Fig. 6B,c,d) and low (LR) (Fig. 6B,e,f) roughness.

Natural and artificial multi-scale surfaces show extreme wetting characteristics that can be exploited for a vast range of applications, including microfluidics, friction reduction and lab-on-a-chip devices^2^,^5^,^6^,^7^. Direct laser micro/nanostructuring has been proven to be a unique tool to fabricate superhydrophobic and water-repellent biomimetic artificial surfaces^4^. In this context, the dual-rough biomimetic surfaces, described above, could provide an appropriate template for the observation of extreme wetting properties. To test this hypothesis the wetting properties of, 16 mm^2^, HR, MR and LR surfaces were evaluated. Following the fs-laser irradiation process, all surfaces showed superhydrophilic behavior (i.e., contact angles CA ~ 0°), which lasted for 6–7 days, depending on the surface structure, while the samples were stored in ambient conditions. The observed decrease of superhydrophilic behavior is attributed to the oxidation of the surface due to irradiation in air environment^34^ and the removal of water molecules^35^. It is observed that the CA progressively increases to a terminal value after 9–10 days of storage in ambient air. This unusual behavior was observed before^34^,^35^ but is not completely understood yet. It may be attributed to the adsorption of hydrocarbon molecules on the irradiated surface from the atmosphere^35^ and/or a change of surface polarity^34^. The study of the water repellent and self-cleaning properties of the fabricated dual-scale surfaces is of special interest and measurements of the water repellent properties are currently under progress.

**Figure 7:**
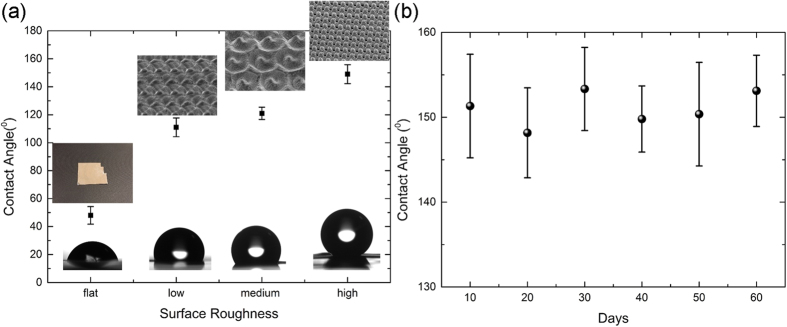
(**a**) Terminated CA values measured on the laser-fabricated low- (LR), medium- (MR) and high- (HR) roughness surfaces, exhibiting the morphology depicted in the insets. The CA of the non-irradiated, flat, Ni surface is also shown for comparison. The respective droplet profiles are also shown as insets; (**b**) Evolution of the terminal CA measured on the laser fabricated on the HR surface as a function of the time of storage in ambient conditions.

The terminated CA measured for water droplets placed on the three different types of dual-rough surfaces (LR, MR, and HR) is presented Fig. 7, along with a reference measurement for the non-irradiated flat surface. All measurements were performed 18–20 days after the fs-laser processing. In particular LR surfaces showed a CA of 111° ± 7°, MR structures exhibited CA of 122° ± 4°, while the HR surfaces had a CA value of 150° ± 5°. The CA of the non-irradiated (flat) surface, 48.6° ± 6°, is also shown for comparison. The CA measurements reveal a remarkable variation among the different surface morphologies, indicating that as the surface roughness increases the CA significantly increases as well. More importantly, the dual-rough HR morphology clearly exhibits a superhydrophobic nature, similar to the behavior exhibited by the natural suprhydrophobic archetype^1^,^2^,^4^, i.e. the *Lotus* leaf surface. It is also similar to the CAs measured on biomimetic surface structures fabricated on Si^4^ and Stainless Steel^33^ using linearly polarized, Gaussian laser beams. Furthermore, the terminal CA measured for the most hydrophobic HR surface was measured to be stable in time upon storage in ambient air for more than two months (Fig. 7b). It can be concluded that ultrafast laser structuring with CV beams can provide a unique tool for controlled structuring at different lengthscales, suitable to obtain biomimetic dual-scale surfaces with extreme wettability. The simplicity of the structuring process, together with the capability to fabricate a practically endless number of complex biomimetic structures, renders this technique very useful for tailoring the wetting response of surfaces in various wettability applications.

## Conclusions

To summarize, we have presented a novel strategy to fabricate highly ordered, multi-directional and complex, biomimetic structures, via exploiting the unique and versatile angular profile of CV fs laser beams. Biomimetic surface structuring was realized through spot-by-spot and large area- scanning of radially and azimuthally polarized beams, giving rise to dual-scale, *Lotus* leaf-like, superhydrophobic surfaces as well as *Shark* skin-like biomimetic morphologies. Although the fabrication of these particular morphologies was demonstrated, laser processing with CV beams has a great potential to provide a plethora of complex structures and biomimetic surfaces. Our approach brings about a new concept in ultrafast laser structuring of materials and can be considered as an emerging laser based fabrication technique, which can be exploited for expanding the breadth and novelty of potential applications. No doubt, our approach requires further development before it can become a competitive technology. However, the wealth of arising possibilities in ultrafast laser based micro and nanofabrication prescribe a future where control of complex surfaces and subsequent functionality can be accomplished with a level of sophistication that we cannot presently envisage.

## Additional Information

**How to cite this article:** Skoulas, E. *et al*. Biomimetic surface structuring using cylindrical vector femtosecond laser beams. *Sci. Rep.* **7**, 45114; doi: 10.1038/srep45114 (2017).

**Publisher's note:** Springer Nature remains neutral with regard to jurisdictional claims in published maps and institutional affiliations.

## Supplementary Material

Supplementary Material
