# Supplementary material for: Biomimetic surface structuring using cylindrical vector femtosecond laser beams
Source: Sci Rep. 2017 Mar 22;7:45114. doi: 10.1038/srep45114 (PMC5361190; doi:10.1038/srep45114)
Supplement: Supplementary Material [file srep45114-s1.pdf]

Biomimetic surface structuring using cylindrical vector femtosecond laser beams

Evangelos Skoulas[1], Alexandra Manousaki[1], Costas Fotakis[1] and Emmanuel Stratakis[1,2]*

[1]Institute of Electronic Structure and Laser (IESL), Foundation for Research and Technology (FORTH), N. Plastira 100, Vassilika Vouton, 70013, Heraklion, Crete, Greece

[2] Materials Science and Technology Department, University of Crete, 71003 Heraklion, Greece

*Corresponding author: stratak@iesl.forth.gr

Supplementary Material

CV & Gaussian beam profiles

Fig 1S presents the two laser beam images and their 3D plots. The laser beam profile was extracted with the use of a CMOS camera close to the focal plane for an S-linearly polarized Gaussian and a radially polarized CV beam. 3D plots were constructed with the pixel intensity values showing the spatial intensity variations for each case.

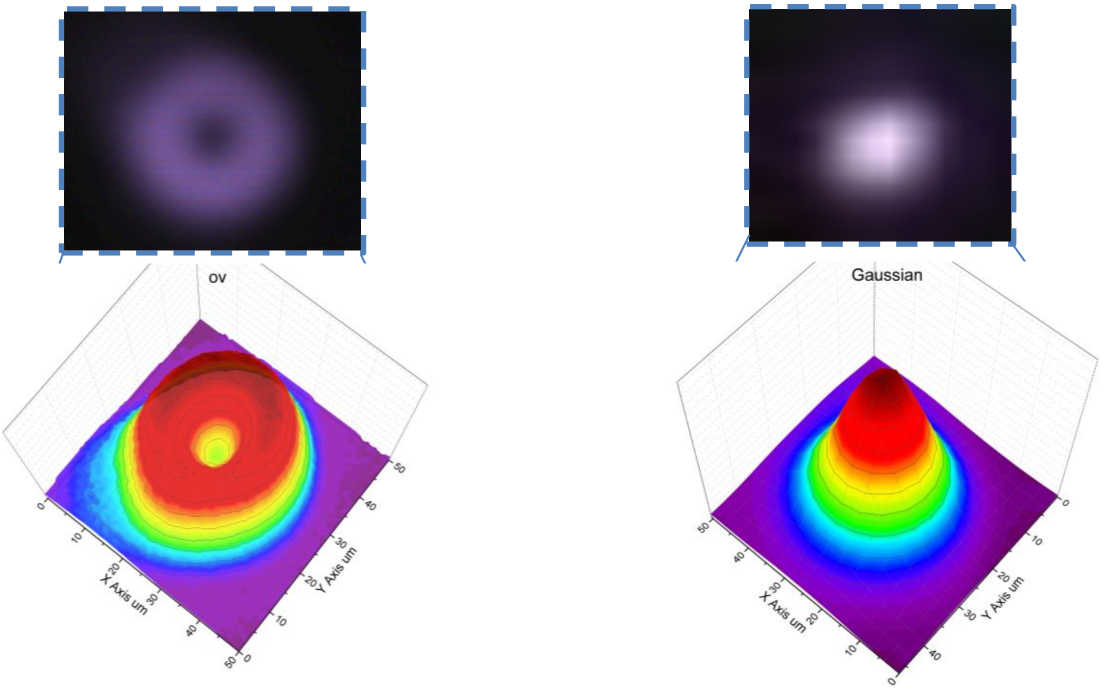

Figure 1S: CV beam (left) and Gaussian beam (right).

Two-dimensional fast Fourier transform on SEM images

In order to be able to extract spatial frequency information a 2D fast Fourier transform (2D-FFT) transform was employed. High-resolution (1280x1024) SEM pictures had been transformed in reverse space images via a 2D-FFT algorithm. The new dimensions of the generated Fourier images are inversely proportional to *x* and *y* dimensions of the original image. Fig.2S presents a typical SEM image of an irradiated laser spot using azimuthal polarization. While Fig.2S(c) shows the corresponding Fourier space image. The orange line represents the direction vertical to the ripple nanostructure. Along this direction the Fourier transformation detects a periodical fluctuation of the frequency intensity. This fluctuation exhibits an average frequency which is inversely proportional to the average ripple period.

In particular, the distance between the centre of Fig.2S (d) and the first peak represents the characteristic frequency *f* of the periodic structure. In order to calculate the periodicity, Λ, of the structures first we calculate the average frequency of *1* and *2* peaks for a vertical as well as a horizontal image cross section (Fig. 2S(d)), and then the average period is given by the relation

$$< \Lambda >= 1/f.$$

Given that the beam can be changed from Gaussian to CV beam, which radically alters the spot surface profile, the LIPSS periodicity values and their relative errors using SEM images of three irradiated spots, produced with identical conditions were calculated. For the estimation of range of frequencies involved into the respective 2D-FFT images, we applied a Lorenzian fit on

both peaks of the cross section and the error of each measurement is calculated using the following relation:

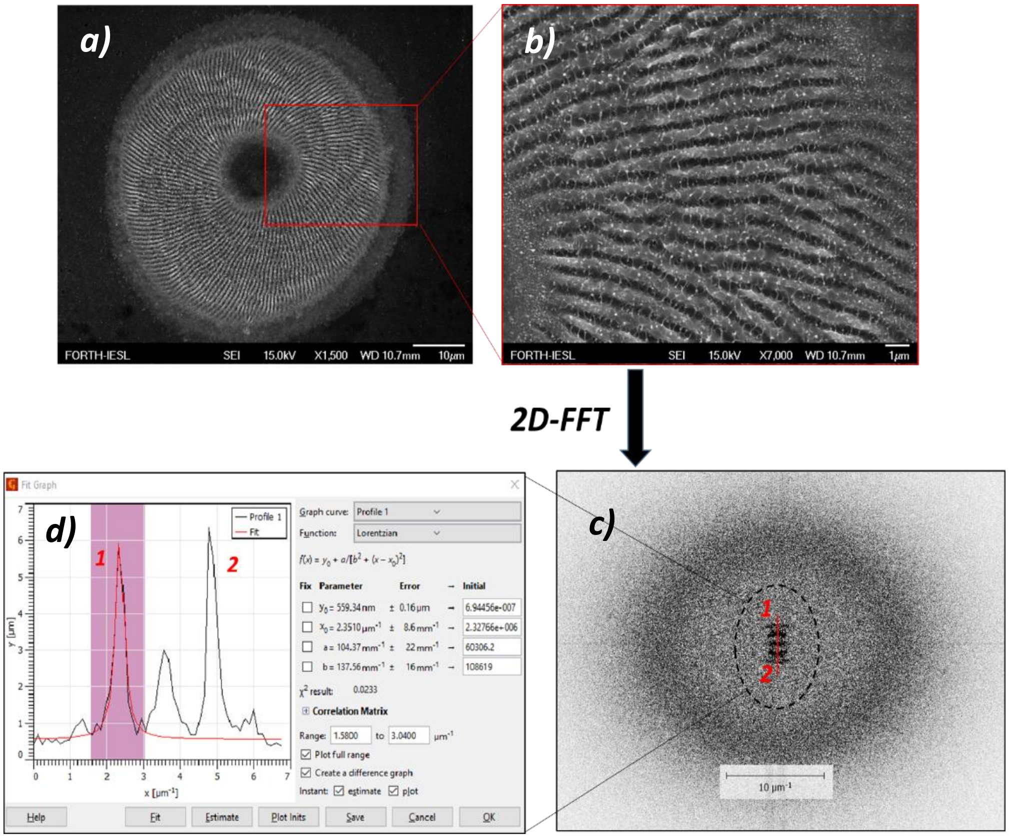

Figure 2S: SEM images of ripple formation on nickel surface, (a) Laser spot, (b) higher magnification of the red box on (a). Image (c) represents the 2D-FFT of the image (b) without the labels on the bottom. The cross section of the black dashed ellipse of the Fourier space image (c) in presented on image (d) with peaks 1, 2 to correspond on the intensity fluctuation of the Fourier image.

$$\boldsymbol{\Delta\Lambda} = \left|-\frac{\mathbf{1}}{\boldsymbol{f}^{\mathbf{2}}}\right| \boldsymbol{\Delta f} \qquad (1)$$

were *Δf* is the mean of the line widths for the two Lorenzian fit curves of the 2D-FFT image profile peaks.

Initial Roughness

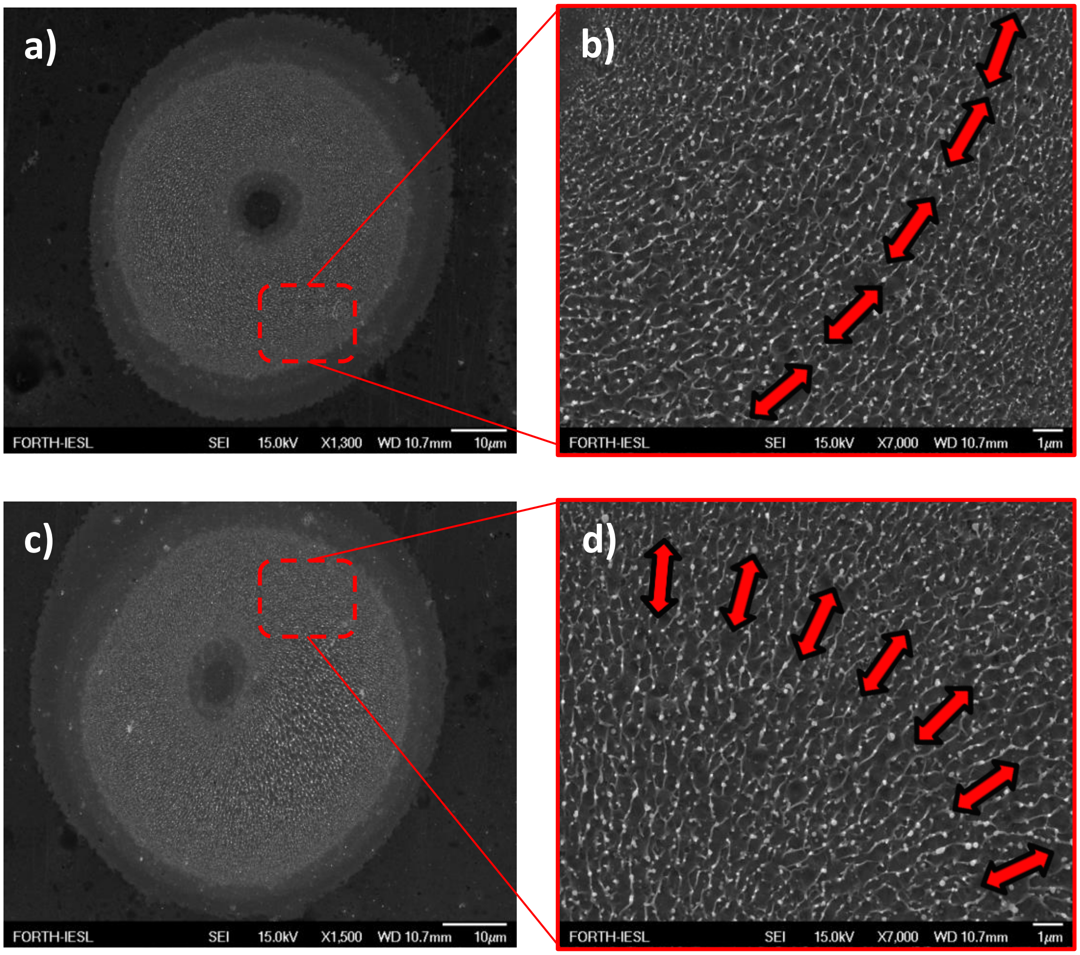

Figure.3S: Figure shows SEM images of 0.49 J/cm^2 with azimuthal (a) 5 pulses, (b) higher magnification of the red dashed area. Radial polarization, (c) 5 pulses, (d) higher magnification of the red dashed area. The red arrows shows the electric field vector distribution.

Results on SEM images showed that at low number of pulses (NP=2-10) and fluence values that range close to the ablation threshold, i.e. 0.17J/cm^2-1.12J/cm^2, the surface shows a mushroom-like nano-roughness, with nanostructures aligned parallel to the incident electric field. Such nanostructures exhibit an average period of 100nm-250nm. Fig.3S shows SEM images of fs laser-iradiated spots at NP=5 and fluence of φ=0.49J/cm^2 with azimuthal (Fig. 3S(a),(b)) and radial polarization (Fig.3S(c),(d)) respectively. Ripple's formation is established following irradiation with NP=10 pulses.

Ripples where observed to always be perpendicular to the incident polarization, regardless the polarization state. Indeed, linear polarization produced ripple structures linearly aligned and perpendicular to the incident electrical field distribution. On the other hand azimuthal and radial polarization showed curved ripple structures, always perpendicularly arranged to the incident electric field. Consequently, ripples produced with azimuthal polarization showed radial orientation, while the ripple structures fabricated with radially distributed electrical field showed concentric circle-like symmetry.

Inducing transparency on thin metallic films

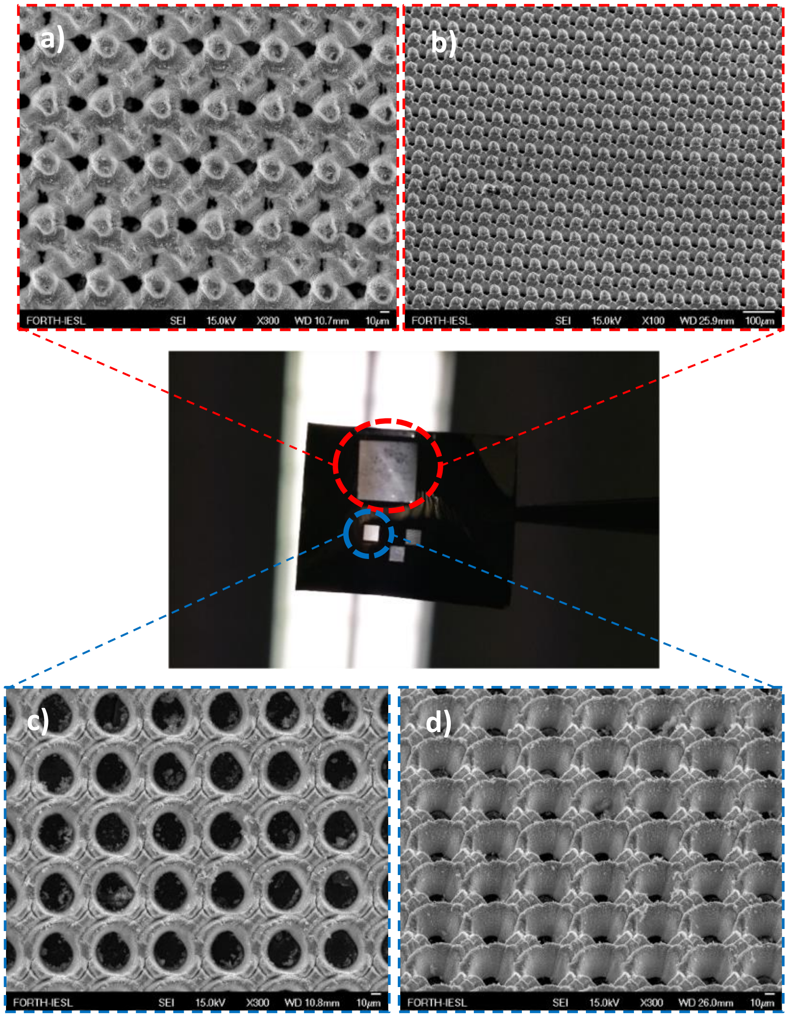

Figure 4S: Photograph of Nickel films under light (middle). SEM images of semi-transparent areas fabricated with intense laser pulses. Images (a) top view (b) tilted at 45^0 represents the red dashed highlighted area fabricated with CV beam and radial polarization at 500 pulses 9,45J/cm^2. Images (c) top view (d) tilted at 45^0 represents the blue dashed highlighted area fabricated with Gaussian beam

Irradiation of thin metallic films, with an average thickness of $d \simeq 100\mu m$, using intense femtosecond pulses, at high fluence values, leads to massive material removal. Due to the small thickness of the film, the material removal could enhance the film transparency. In this context, we have conducted a series of experiments aiming at altering the layer thickness and fabricate laser structured transparent metallic membrane areas.

For the fabrication of the transparent membrane-like surfaces we used linearly polarized Gaussian as well as radially polarized CV beams. Typical SEM images are presented in Fig.4S. In the same Figure one can observe the treated areas of 4x4mm and 1x1mm under normal light illumination conditions. All laser treated areas show a significantly stronger light transmission compared to the untreated ones.

Surface morphological profile

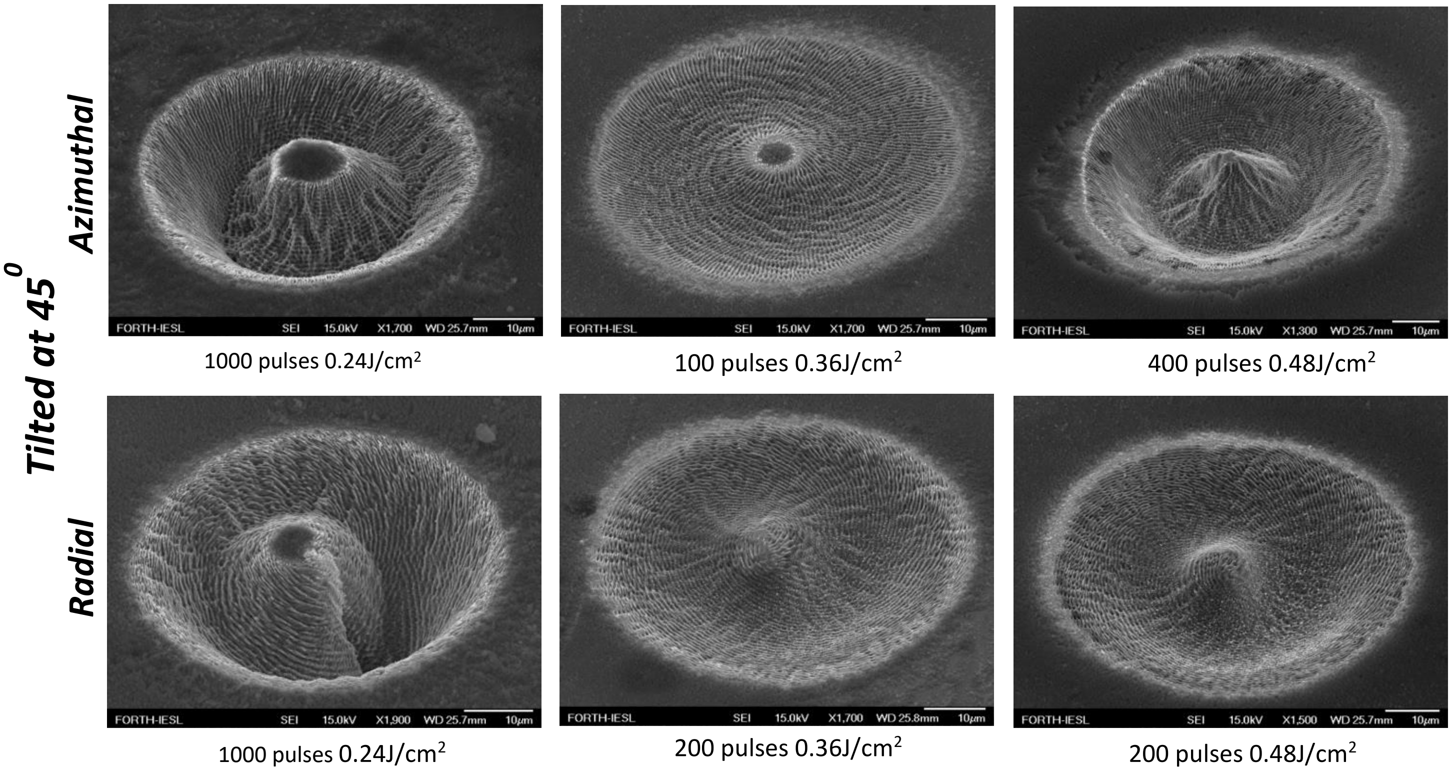

Figure 5S: SEM images tilted at 45^0 with the morphological profile on Ni surfaces after fs-irradiation with variable fluence and NP values.

Parametric with CV beam Line Scanning

SEM Radial & Azimuthal lines at variable scan velocities

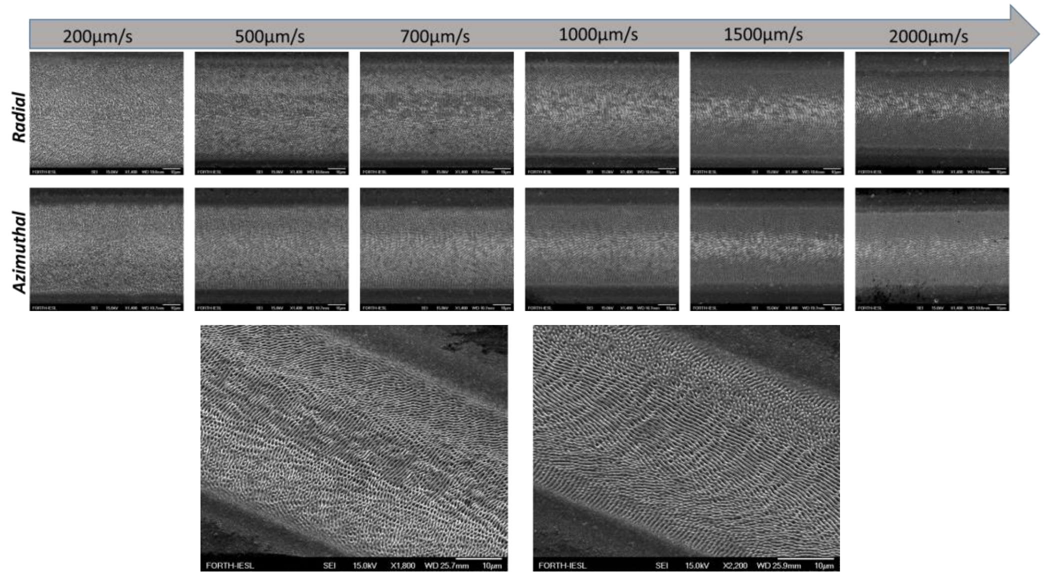

Figure.6S: Line scans of φ=0.24cm^2 for 5 different scan velocity values.

Ablation threshold fluence estimation

Parametric study was initially conducted by performing single shot irradiations (NP=1), on nickel surfaces, at different fluences. The estimation of the ablation threshold fluence, was found to be at φ_{th}=0.17J/cm^2 and 0.99J/cm^2 for the radially and azimuthally polarized CV beams

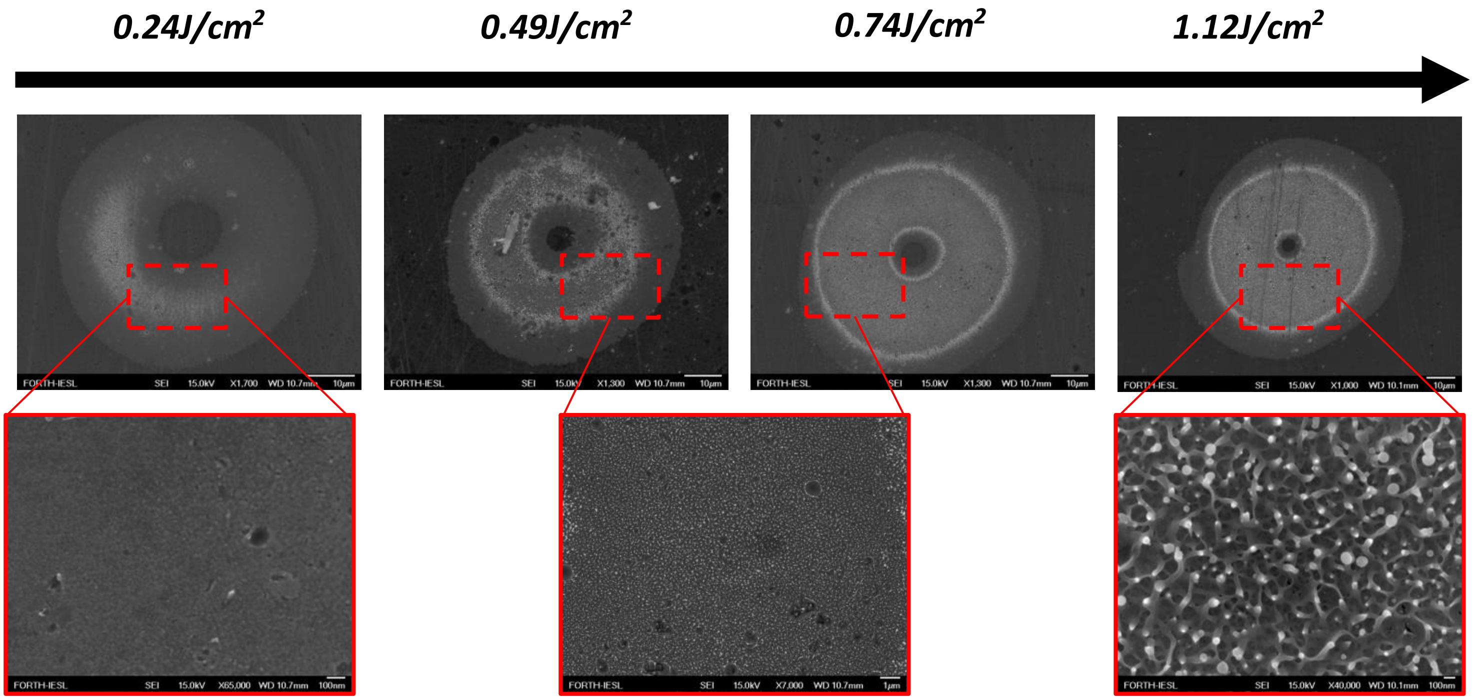

Figure 7S: SEM images are presented from single shot (NP=1) irradiations of Nickel targets with azimuthally polarized laser pulses, for the estimation of the ablation threshold fluence area. The red outlined images represent higher magnification of the red dashed area.

respectively. It was aslo found that irradiation with fluence 0.11J/cm^2 ≤ φ ≤ 0.17J/cm^2 can cause phase transition and give rise to a sort of surface roughness due to rapid resolidification of the melted material.

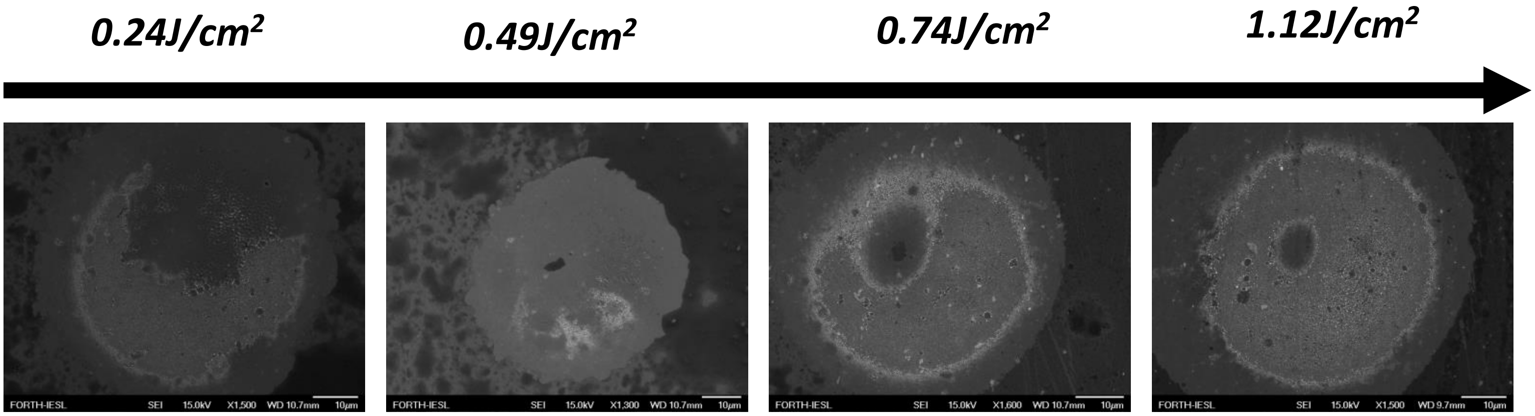

Figure 8S: SEM images are presented from single shot (NP=1) irradiations of Nickel targets with radially polarized laser pulses, for the estimation of the ablation threshold fluence area.

Consequently, we can identify two fluence value regimes, the sub-ablation one ($0.11 J/cm^2 \leq \varphi \leq 0.17 J/cm^2$) and the above-ablation one with fluences of $0.17 J/cm^2$ and higher. Fig.7S and Fig.8S present SEM images with single shot irradiation of Nickel surfaces with azimuthal and radial polarization beams respectively. It was observed that sub-ablation fluence values could not produce LIPSS at low pulse numbers, while above-ablation fluence values strongly ablate the surface for high number of pulses. In view of this we have decided to work at a fluence area that can give rise to LIPSS for both low and high number of pulses.
